# Amino-Pyrazoles in Medicinal Chemistry: A Review

**DOI:** 10.3390/ijms24097834

**Published:** 2023-04-25

**Authors:** Matteo Lusardi, Andrea Spallarossa, Chiara Brullo

**Affiliations:** Department of Pharmacy (DIFAR), Section of Medicinal Chemistry, University of Genoa, Viale Benedetto XV 3, 16132 Genoa, Italy

**Keywords:** pyrazole, aminopyrazole, p38MAPK, medicinal chemistry, biological properties

## Abstract

A pyrazole nucleus is an easy-to-prepare scaffold with large therapeutic potential. Consequently, the search for new pyrazole-based compounds is of great interest to the academic community as well as industry. In the last ten years, a large number of papers and reviews on the design, synthesis, and biological evaluation of different classes of pyrazoles and many pyrazole-containing compounds have been published. However, an overview of pyrazole derivatives bearing a free amino group at the 3, 4, or 5 position (namely, 3-aminopyrazoles, 4-aminopyrazoles, and 5-aminopyrazoles, respectively) and their biological properties is still missing, despite the fact that aminopyrazoles are advantageous frameworks able to provide useful ligands for receptors or enzymes, such as p38MAPK, and different kinases, COX and others, as well as targets important for bacterial and virus infections. With the aim to fill this gap, the present review focuses on aminopyrazole-based compounds studied as active agents in different therapeutic areas, with particular attention on the design and structure-activity relationships defined by each class of compounds. In particular, the most relevant results have been obtained for anticancer/anti-inflammatory compounds, as the recent approval of Pirtobrutinib demonstrates. The data reported here are collected from different databases (Scifinder, Web of Science, Scopus, Google Scholar, and Pubmed) using “aminopyrazole” as the keyword.

## 1. Introduction

The pyrazole heterocyclic ring represents an important building block in different areas of organic and medicinal chemistry [1,2] as well as industrial and agricultural applications. Different pyrazole derivatives are used in supramolecular and polymer chemistry, in the food industry, as cosmetic colourings, and as UV stabilisers, while other pyrazoles have liquid crystal properties [3]. However, pyrazole compounds are rarely found in natural products, which is probably related to the difficulty of N-N bond formation by living organisms [4].

**Figure 1:**
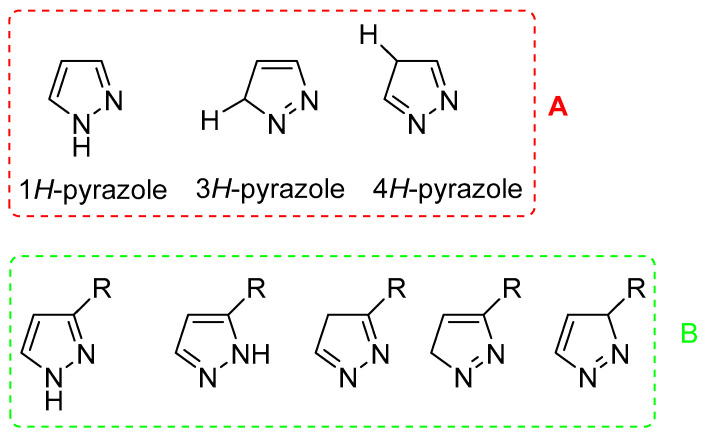
(**A**) Unsubstituted pyrazoles tautomerism. (**B**) Mono-substituted pyrazoles tautomerism.

Pyrazole-containing molecules display a broad range of biological activities, including anti-inflammatory [5,6], anticonvulsant [7], anticancer [8,9], antiviral [10], antidepressant [11], analgesic [12], antibacterial [13,14,15], antifungal [16], and selective enzyme inhibition [16,17]. Moreover, several pyrazole compounds are currently used in clinics as anti-inflammatory and analgesic (Celecoxib, Tepoxalin, and Betazole), anticancer (Crizotimib), antiobesity (Surinabant and Difenamizole), antidepressant, and tranquilliser (Fezolamine and Mepiprazole) drugs, thus confirming the pharmacological value of this heterocycle. The pharmacological properties of its nucleus are related to its particular chemical behaviour; pyrazole presents a nitrogen atom 1 (N1), named “pyrrole-like” because its unshared electrons are conjugated with the aromatic system, and a nitrogen atom 2 (N2), named “pyridine-like” since the unshared electrons are not compromised with resonance, similarly to pyridine systems. For this reason, pyrazole can react with both acids and bases [18]. An additional structural characteristic of pyrazole is its prototrophic tautomerism; in fact, three tautomers are possible in unsubstituted pyrazoles (Figure 1A), while five tautomers can exist in mono-substituted pyrazoles (Figure 1B) [19].

**Figure 2:**
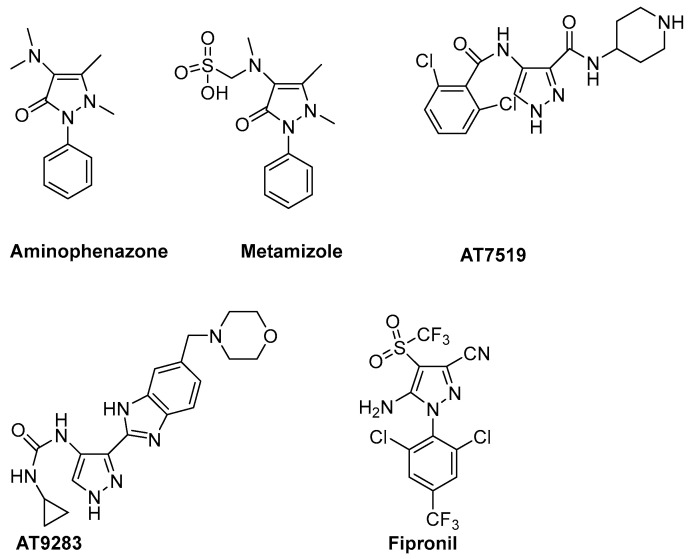
The general structure of Aminophenazone, Metamizole, AT7519, AT9283, and Fipronil.

The functionalisation of the pyrazole nucleus with amino substituents in different positions has led to multifunctional pharmacologically active compounds [20,21]; in fact, aminopyrazoles (APs) represent a versatile and very useful framework in drug discovery [22]. Some APs have the free amino group (NH_2_), others bear a substituted amino group, and in some other derivatives, the amino function is part of other heterocycles. Several bioactive pyrazoles (e.g., Aminophenazone and Metamizole, Figure 2) present an amino group at position 4; this chemotype is shared by different drug candidates currently in clinical trials as potent inhibitors of several cyclin-dependent kinases (CDKs) AT7519 (Figure 2; five different clinical trials) [23] and AT9283 (Figure 2), known as a multitargeted kinase inhibitor with potent Aurora kinase inhibition [24] (five clinical studies). In addition, Fipronil (Figure 2) [16] is a highly substituted 5-aminopyrazole with insecticidal activity. In fact, this compound is able to reversibly block pest GABA-A receptors and disturb the activity of the insect’s central nervous system, causing hyperexcitation of the nerves and muscles of the contaminated insects. Fipronil insecticide is commonly used for the protection of crops and ornamental plants against herbivorous insects and mites, urban pest control, fish farming, and veterinary applications.

Due to the importance of this scaffold in medicinal chemistry research, a lot of reviews and articles have reported different summaries regarding the chemical synthesis and pharmacological properties of pyrazole derivatives, with APs being the most studied chemotype [25,26]. Particular attention has also been paid to pyrazole synthesis methods [2,25,26]. Collectively, anticancer and antimicrobial properties have emerged as the most frequent biological uses reported for the pyrazole scaffold [16,27,28].

**Figure 3:**
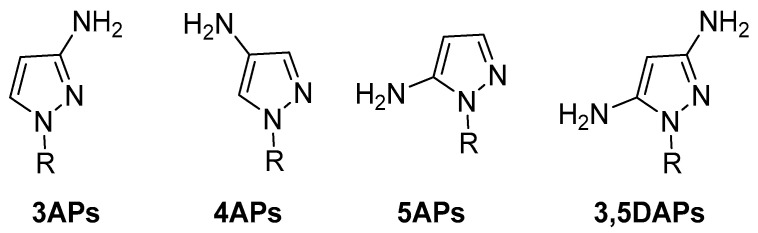
The general structure of 3APs, 4APs, and 5APs.

In view of pyrazole’s high potential behaviour, in the current review, we focused our attention on the AP scaffold, which represents one of the most studied moieties in medicinal chemistry, both in academia and in industry. This review focuses on AP compounds presenting a free amino group (hydrogen bond donor) which is responsible for the formation of numerous interactions with different targets. We, therefore, identify 3-aminopyrazoles (3APs), 4-aminopyrazoles (4APs), and 5-aminopyrazoles (5APs) (Figure 3) which, during the last 20 years, have been extensively studied in various fields of medicinal chemistry. In addition, some examples of 3,5-diaminopyrazoles (3,5DAPs) were reported.

## 2. 3-Aminopyrazoles (3APs)

As reported below, compounds characterised by a pyrazole scaffold substituted with an amino group in position 3 (3APs) are largely reported as anticancer and anti-inflammatory agents but are also known for their anti-infective properties.

### 2.1. Anti-Infective 3APs

Regarding anti-infective activity, 3APs have been investigated as antibacterial and antiviral agents; in some cases, the 3AP scaffold has been incorporated into a more complex structure.

**Figure 4:**
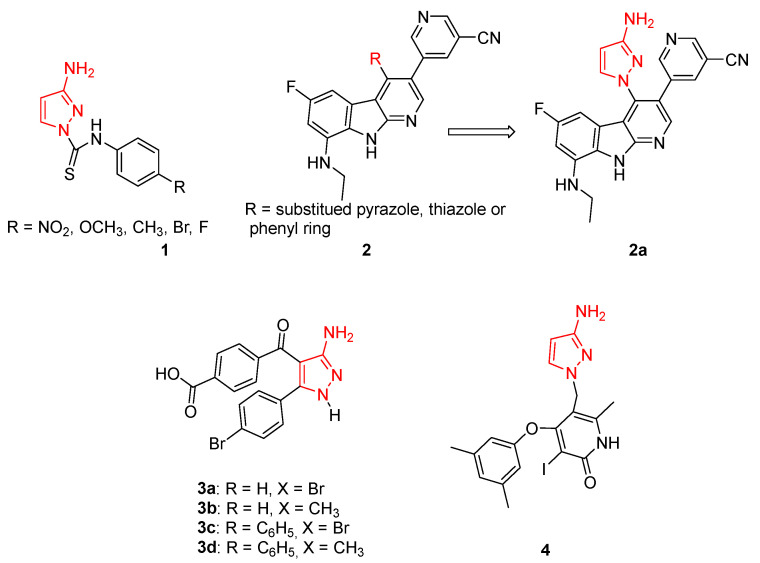
3APs as anti-infective agents. The 3-aminopyrazole portion is highlighted in red.

In detail, Delpe-Acharige and coll. reported a series of 3APs bearing a thiourea moiety in N1 (compounds **1**, Figure 4) with sub-micromolar activity against *Methicillin-sensitive Staphylococcus aureus* (MSSA) and *Methicillin-resistant S. aureus* (MRSA) in the presence of bioavailable copper. This study clearly established the suitability of using pyrazolyl thioureas for the treatment of these types of infections [29].

In 2020, some authors from Roche Pharma Research and Early Development identified a small library of pyrido[2,3-*b*]indole **2** (Figure 4) able to block Gram-negative strains, targeting both DNA Gyrase and Topoisomerase IV. Among all the synthesised compounds, derivative **2a** (Figure 4), characterised by a 3AP substituent on a pyrido-indole scaffold, exhibited MIC values of 0.125 and 8 mg/mL against *S. aureus* and *E. coli*, respectively [30].

In the same year, Fahim and coll. synthesised a series of 3APs as intermediates for the synthesis of novel pyrazolo[1,5-*a*]pyrimidine derivatives. All the newly isolated compounds were screened on a Well Diffusion Assay to evaluate their antimicrobial activity against three bacteria strains (*B. subtilis*, *S. pneumoniae*, and *E. coli*) and three fungi (*A. flavus*, *S. racemosum*, and *G. candidum*). The 3APs **3a**–**d** (Figure 4) showed high activity against both bacteria and fungi strains (inhibition zone diameter > 15 mm), highlighting the promising properties of this class of compounds as antibacterial agents [31].

In 2009, researchers from TIBOTEC (France), identified some 4-aryloxy-3-iodopyridin-2(1*H*)-ones that have been evaluated as anti-HIV inhibitors. The authors investigated different substituents at the 5-position of this scaffold, including one compound characterised by a 3AP moiety in the 4 position (derivative **4**, Figure 4) which showed potent HIV-1 reverse transcriptase inhibitory properties against both the wild-type enzyme and simple/double mutated forms [32].

### 2.2. Anticancer and Anti-Inflammatory 3APs

**Figure 5:**
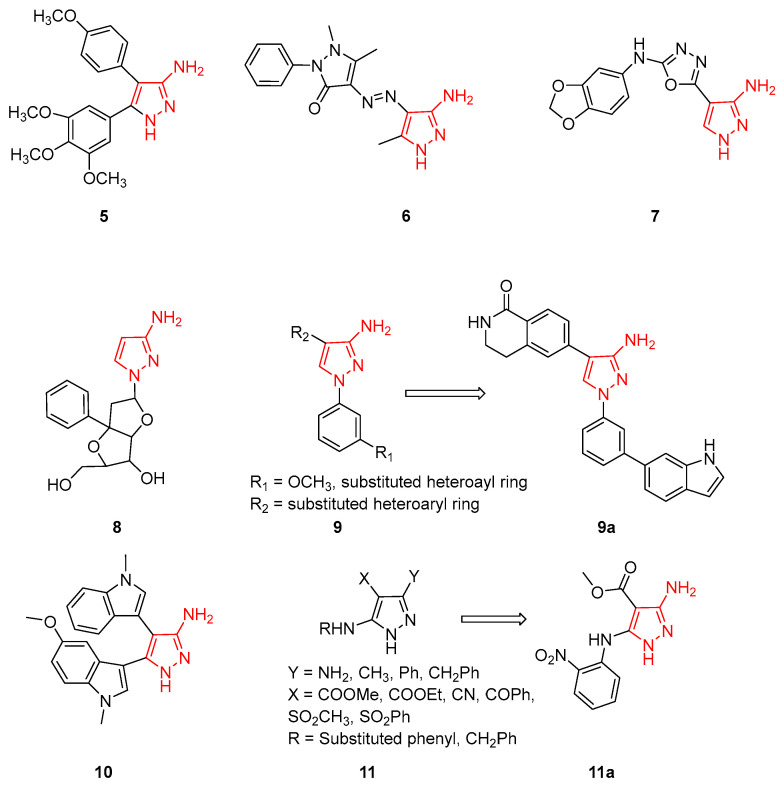
3APs as anticancer and anti-inflammatory agents. The 3-aminopyrazole portion is highlighted in red.

The most active 3AP compounds with antitumour and anti-inflammatory activities are characterised by unsubstituted scaffolds on N1 and often bear bulky aromatic rings on C4. In detail, a series of 4,5-diaryl-3APs were synthesised using Combrestatin A-4 (a well-known microtubule inhibitor) as the lead compound. The new 3APs were tested for their cytotoxic activity in vitro against five human cancer cell lines (i.e., K562, ECA-109, A549, SMMC-7721, and PC-3) and the different derivatives showed potent cytotoxicity against all the tested cell lines, with IC_50_ values in the low micromolar range. Compound **5** (Figure 5) was identified as the most interesting derivative, with IC_50_ values in the 0.08–12.07 mM range. Additional biological tests indicated that Compound **5** was a potent inhibitor of tubulin polymerisation, arresting the cell cycle in the G2/M phase. In addition, in order to investigate the binding pose of this 3AP to the colchicine binding site, docking simulations were carried out using the crystal structure of the tubulin–colchicine complex. This study evidenced that:

✓ The trimethoxyphenyl moiety and 4-methoxyphenyl moiety of Compound **5** are positioned in the hydrophobic pocket between Alaβ250-Alaβ316 and Valα181-Metβ259, respectively;
✓ The trimethoxyphenyl moiety is situated in close proximity to Cysβ241;
✓ The oxygen atom of the 4-methoxy substituent forms a hydrogen bond with the thiol group of Cysβ241;
✓ The pyrazole NH group establishes one hydrogen bond with the Alaβ250 backbone NH functionality;
✓ The hydrogen atom of 3-NH_2_ forms another hydrogen bond with the NH of Asnα101.

Collectively, these results confirmed the experimental data and demonstrated that Compound **5** could represent an interesting chemotype for anticancer activity, suggesting that the 4,5-diaryl-3APs could be considered as Combrestatin A-4 mimetics [33,34].

Simultaneously, other researchers, using different synthetic approaches, synthesised and evaluated the antitumor and antioxidant activity of some novel pyrazolo-triazines to verify their pharmacological activity [35]. All compounds were evaluated for their in vitro anticancer effect with the standard MTT method against a panel of four human tumour cell lines, including hepatocellular carcinoma (HepG2), lung fibroblasts (WI 38), and breast cancer (MCF-7) with 5-Fluorouracil (5-FU) used as the reference compound. In addition, all derivatives were tested to evaluate their cytotoxicity against a well-known established model of Ehrlich ascites cells (EAC) in vitro [36] and for their antioxidant activity [37]. Within this library of pyrazolo-triazines, 3AP **6** (Figure 5), used as an intermediate for the synthesis of different pyrazolo-triazines, showed IC_50_ values ranging from 73 to 84 mg/mL against different cancer cell lines as well as some antioxidant activity. In conclusion, the authors demonstrated that the AP moiety enhances the antioxidant properties of different heterocycle systems [35].

Differently, other authors from deCODE Chemistry (Chicago, IL, USA), during the development of variously substituted 1,3,4-oxadiazole derivatives to obtain anti-proliferative and antimitotic agents with microtubule destabilising activities, reported that the introduction of a 1,3,4-oxadiazole scaffold on a 3AP substituent is detrimental for anticancer activity, as demonstrated by biological activity Compound **7** (Figure 5, EC_50_ values > 50 mM) [38].

In 2008, Japanese researchers reported a 3AP linked to a nucleoside analogue, with the purpose of obtaining a triplex-forming oligonucleotide (named TFOs) able to bind the major groove of the DNA duplex and therefore identify novel genomic tools. Nucleoside-3AP **8** (Figure 5) was able to recognise the CG interrupting site, but additional studies are necessary to validate its pharmacological activity [39].

3APs have been also studied as inhibitors of different kinases involved in the inflammatory process, obtaining interesting results. In fact, in 2010, researchers from Novartis Institutes (Basel, Switzerland) applied a scaffold hopping strategy and identified some differently substituted 3APs **9** (Figure 5) as MK2-inhibitors. MK2 is a direct downstream kinase substrate of p38 mitogen-activated protein kinases (MAPKs) that plays a crucial role in the signalling and synthesis of TNFa, having a central role in inflammation and auto-immune diseases. The new derivatives also were shown to inhibit the intracellular phosphorylation of HSP27 and the LPS-induced TNFa release in cells. 3AP **9a** (Figure 5), bearing an additional indole moiety on the N1 pyrazole, emerged as the most active compound of the series. Furthermore, the compound also inhibited LPS-induced TNFa release in mice and X-ray crystallography studies of the MK2/**9** complex evidenced an unusual binding conformation with the indole ring inserted in a new ligand-induced hydrophobic pocket behind the MK2-hinge region. In detail, the indole-NH group forms an additional hydrogen bond interaction with the backbone carbonyl of Phe90, highlighting the importance of a hydrogen donor at this position. As expected, the 3AP moiety binds to the hinge region of MK2. All these interactions seem to improve the binding to MK2 and also kinase selectivity. In a summary, the 3AP scaffold was confirmed as a new interesting portion for the identification of novel MK2-inhibitors able to treat different inflammatory disorders [40].

Ten years later, 3APs were studied as inhibitors of GSK-3β, a serine/threonine kinase that plays critical roles in multiple cellular functions in the central nervous system and is considered a key player in Alzheimer’s disease (AD) pathophysiology. Most synthesised 3APs possess GSK-3β inhibitory activities, with IC_50_ values in the micromolar ranges, and bisindole-substituted 3AP **10** (Figure 5) displayed moderate GSK-3β inhibition (IC_50_ = 1.76 ± 0.19 μM). Furthermore, the compounds influenced LPS-induced glial inflammation in BV-2 cells and glutamate-induced oxidative neurotoxicity in HT-22 cells. Additional in vivo studies confirmed the anti-inflammatory effect of **10** that proved to reduce microglial activation and astrocyte proliferation in the brain of LPS-injected mice. Overall, this study evidenced specifically that bisindole-substituted 3AP could be a useful prototype for the discovery of novel therapeutic agents to tackle AD and other GSK-3β-associated complex neurological syndromes [41].

Finally, more recently, Lusardi and coworkers developed a new regioselective procedure to synthesise novel highly functionalised 3APs **11** (Figure 5) [42]. All the prepared derivatives were tested by an MTT assay on a panel of tumour cell lines and against normal fibroblast to evaluate their antiproliferative activity. Methyl 3-amino-5-[(2-nitrophenyl)amino]-1*H*-pyrazole-4-carboxylate (compound **11a**, Figure 5) displayed good inhibition of the proliferation on HepG2 (liver cancer cells) and HeLa (cervical cancer cells) with mean growth percentage values of 54.25% and 38.44%, respectively. On the other hand, the compound was inactive against normal fibroblasts GM-6114 (growth percentage = 80.06%), showing no toxicity to healthy cells. Interestingly, inserting different alkyl and aryl moieties at position N1 of the pyrazole led to a loss of antiproliferative activity against all tested cell lines [43].

This evidence highlighted the importance of unsubstituted N1 nitrogen for the cytotoxic/antiproliferative activity of this class of compounds, as demonstrated by the activity of compounds **5**–**7**, and **11**. On the other hand, when a more embedded substitute was inserted in this position, as in Compound **9**, anti-inflammatory activity was reported.

## 3. 4-Aminopyrazoles (4APs)

According to the literature data so far available, 4AP compounds showed reduced anti-inflammatory and anticancer activity in comparison with their 3AP and 5AP isomers but attracted some attention as anticonvulsants, colouring, and antioxidant agents. Due to the variety of these biological properties, SAR considerations are difficult to define.

**Figure 6:**
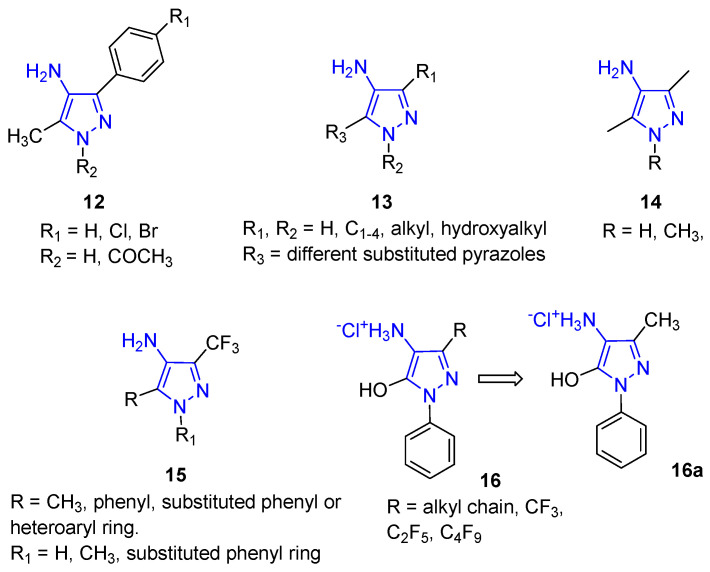
4APs reported as pharmacological agents. The 4-aminopyrazole portion is highlighted in blue.

In detail, since 1970, molecules with a pyrazole structure carrying the amino group in position 4 (4APs) have been studied for their interesting anticonvulsant properties. Compound **12** (Figure 6) is characterised by a methyl group at position 3 and by a substituted phenyl ring at position 5; research evidenced some anticonvulsant activity, and the authors demonstrated the importance of the distance between the exocyclic NH_2_ group and the endocyclic NH for this type of pharmacological activity [44]. More recently, interesting results have been obtained for similar 4AP analogues tested as anticonvulsant agents [45].

Different 4APs, characterised by an additional pyrazole nucleus on the N1 position (**13**, Figure 6), were patented as keratin dyeing [46], evidencing the importance of this scaffold in industry as well.

4APs were also prepared as intermediates in the synthesis of a large library of thiourea and urea derivatives as anticonvulsant agents. Unfortunately, Compound **14** (Figure 6) and related urea and thiourea derivatives were shown to be poorly active in pentylenetetrazole-induced seizure (PTZ) and maximal electroshock tests. At the same time, anti-HIV pharmacological evaluation was also carried out, obtaining negative results [47].

However, the interest in this scaffold has grown in recent years, highlighting the different antioxidant properties of this chemotype.

In 2020, a simple and efficient method for the synthesis of different pyrazole derivatives **15** (Figure 6), including 4APs bearing various substituents at positions 1 and 5, was developed. The prepared compounds were tested in vitro for their tuberculostatic, antibacterial, antimycotic, antioxidant, and cytotoxic activities; additionally, the analgesic and anti-inflammatory properties of the compounds were evaluated in vivo. According to the calculated ADME parameters, all obtained pyrazole derivatives evidenced favourable pharmacokinetic profiles. In detail, synthesised compounds revealed multiple pharmacological activities depending on the nature of their peripheral substituents at the pyrazole core. 4APs **15** did not show antibacterial and antimycotic activity but revealed anticancer activity against HeLa cells and moderate toxicity on human dermal fibroblasts (HDF). Moreover, the N-unsubstituted 4APs and their hydrochlorides showed good antioxidant properties whereas their N-substituted analogues were inactive or significantly less active. In addition, the introduction of thienyl moiety at position 5 significantly increased the acute toxicity of 4APs. The best analgesic activity was evidenced for 4APs having a phenyl fragment at position 5. Interestingly, among all the series of tested compounds, only 4-amino-5-phenylpyrazoles had appreciable anti-inflammatory activity. The developed SARs profile pointed to the 4-amino-3-trifluoromethyl-5-phenylpyrazoles as the lead structure for the development of new pharmacologically active compounds [48].

More recently, the same authors reported the antioxidant activity of different Edaravone analogues characterised by the 4AP scaffold (**16**, Figure 6). These derivatives, obtained by the reduction of 4-hydroxyiminopyrazol-5-ones, showed pronounced antioxidant activity in different assays (namely, the ABTS, FRAP, and ORAC tests), with the 4-amino-3-methyl-1-phenylpyrazol-5-ol hydrochloride (**16a**, Figure 6) derivative being the most active. Additional investigations confirmed the promising properties of **16**, which was proposed as a lead structure for developing novel therapeutic drug candidates for treating oxidative stress-related diseases. Additional chemical modifications, such as conjugation to an anticholinesterase fragment, could be performed to obtain multifunctional drugs for treating neurodegenerative diseases [49].

## 4. 5-Aminopyrazoles (5APs)

The substitution of the amino group in position 5 of the pyrazole ring is largely reported in the literature due to the high versatility of this class of compounds in the medicinal chemistry field. As reported below, these derivatives have been used as kinase inhibitors (particularly p38MAPK and Bruton kinase inhibitors), anticancer, antibacterial, antimalarial, and anti-inflammatory agents.

### 4.1. p38 Inhibitors 5APs

The MAPK family regulates a variety of cellular responses such as proliferation, differentiation, gene expression, cell survival, and apoptosis through the transduction of extracellular signals [50]. p38, together with JNK and ERK, belongs to the MAPK family, and it is mainly involved in the regulation of pro-inflammatory cytokines such as TNF-a, IL-1, and IL-6 [51]. For all these reasons, p38MAPK inhibitors have been largely studied as anticancer [52,53] and anti-inflammatory [54,55] agents. Particularly, 5-pyrazolyl ureas have been widely studied as p38MAPK inhibitors, but unfortunately, their therapeutic use is precluded by different side effects and ineffectiveness in clinical studies [19]. For these reasons, the urea moiety on position 5 has been deleted and different 5APs have been studied.

**Figure 7:**
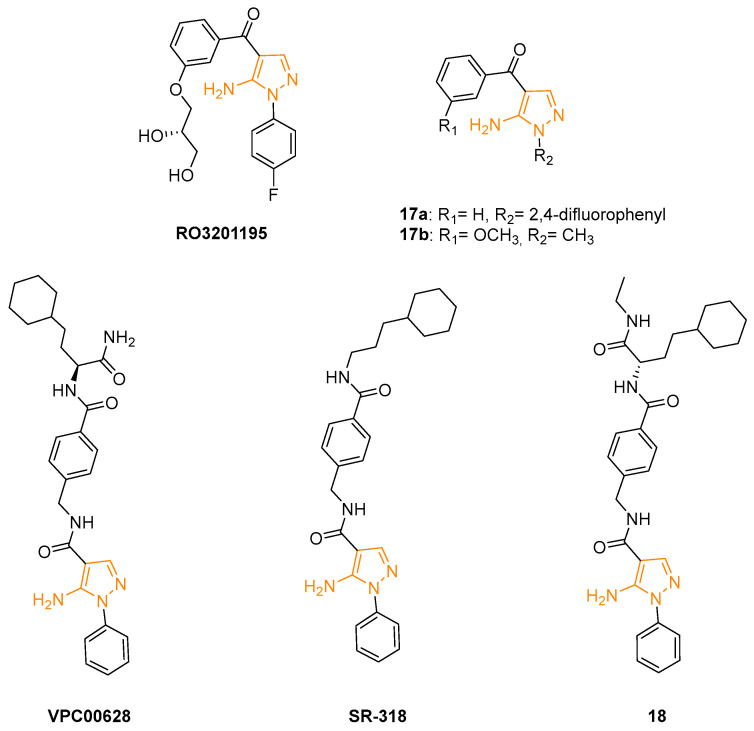
5APs reported as p38MAPK inhibitors. The 5-aminopyrazole portion is highlighted in orange.

In 2006, Goldstein and coworkers designed and synthesised a new series of highly selective p38MAPK inhibitors with a 5-amino-*N*-phenyl-1*H*-pyrazol-4-yl-3-phenylmethanones scaffold. X-ray crystallography of these new derivatives bound in the ATP binding pocket of unphosphorylated p38a was used to optimise the potency and physicochemical properties of the series. The addition of a 2,3-dihydroxypropoxy moiety on the C4 phenyl ring led to the isolation of compound RO3201195 (Figure 7), characterised by a higher selectivity on p38a and excellent drug-like properties, including high oral bioavailability. This 5AP molecule displayed an IC_50_ of 0.7 ± 0.1 mM on p38a, good inhibition of TNFa production in a human monocytic cell line (THP1), and a reduction in IL-1b in a mononuclear cell fraction isolated from HWB. Compound RO3201195 was also tested in several acute inflammatory models in rats to evaluate its efficacy in vivo. The obtained results showed a significant dose-dependent inhibition of serum TNFa and IL-6 (IC_50_ values of 0.2 and 0.3 mM, respectively) [56]. A few years later, Bagley and coll. proposed a new synthetic method for the preparation of RO3201195 and tested the compound on Werner syndrome (WS) cells. High levels of phosphorylated p38a are expressed in proliferating WS cells, demonstrating that p38MAPK pathway inhibition could potentially interfere with the pathology. The results obtained in hTERT-immortalised HCA2 cells and primary WS cells treated with RO3201195 confirmed this hypothesis; in fact, this compound showed excellent selectivity for p38aMAPK over JNK and slowed down the accelerated aging of WS cells in culture [57].

The same research group extended the SARs of previously synthesised 5APs and tested the newly obtained derivatives on human hTERT-immortalised HCA2 dermal cells to evaluate their ability to inhibit p38MAPK. The four compounds that displayed comparable or slightly improved potency over RO3201195 were tested on WS cells. Particularly, the 2,4-difluorophenyl substituent of compound **17a** (Figure 7) seemed to increase the activity compared to other derivatives, as well as the methyl substitution on N1 of compound **17b** (Figure 7). These two molecules were selected as lead compounds but, despite their good p38a inhibition, they did not improve the efficacy and bioavailability of RO3201195 in in vivo evaluations [58]. Other experiments were carried out on WS cells with RO3201195 and its analogues **17a** and **17b**. The results definitively confirmed that the proliferation of pathological cells is linearly correlated to p38 phosphorylation and that these 5APs significantly interfere with the protein expression and consequently with cell growth. Furthermore, the derivatives showed a better selectivity on p38 in comparison with the reference compound SB203580 which seemed to interfere in the same way with both p38 and JNK MAPK [59].

**Figure 8:**
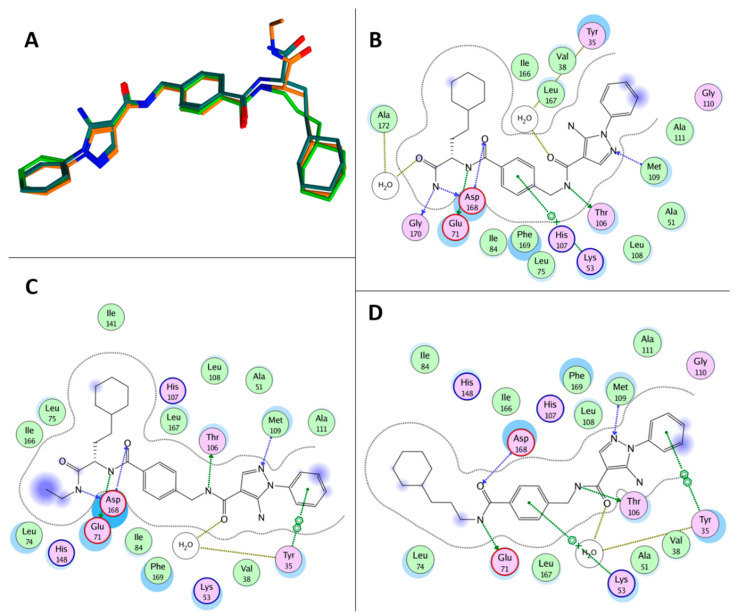
(**A**) Superposition of the bioactive conformations of VPC00628 (dark green), SR-318 (green), and **18** (orange) as observed in the corresponding crystallographic complexes with p38MAPK. (**B**) Ligplot of the interactions between VPC00628 and p38MAPK. (**C**) Ligplot of the interactions between **18** and p38MAPK. (**D**) Ligplot of the interactions between SR-318 and p38MAPK.

In 2016, the screening of a DNA-encoded small molecule library allowed the identification of the highly specific and potent (IC_50_ = 7 ± 0.9 nM) p38aMAPK inhibitor VPC00628 (Figure 7). This compound shares the N1 phenyl ring with RO3201195 and bears an amide function (rather than a keto group) on the C4 position. X-ray crystallography studies (PDB code: 5LAR) indicated that VPC00628 interacts with the ATP binding site of p38aMAPK, inducing a strong distortion of the P-loop. Interestingly, the ligand assumes an alternative binding mode as it lacks the key features of known kinase inhibitors such as a typical hinge binding motif. VPC00628 showed excellent shape complementarity and formed several specific polar interactions (Figure 8), assuming a canonical inactive type-II (‘DFG-out’) binding mode. This specific interaction with the inactive form of the kinase seems fundamental to increasing the potency and the selectivity of the inhibitor [60].

Röhm and coworkers synthesised a new series of VPC00628 analogues, trying to improve the pharmacological properties of the parent compounds. Using a systematic combinatorial synthetic approach, they isolated and identified SR-318 (Figure 7), a 5AP characterised by a more hydrophobic amide moiety. The crystal structure of the SR-318/p38MPAK complex (PDB code: 6SFO; Figure 8) provided the structural basis for the excellent potency and selectivity for p38a/b (IC_50_a = 5 nM and IC_50_b = 32 nM) of the identified derivative. Moreover, SR-318 showed a better metabolic degradation profile in comparison with the reference drug VPC00628. To test the in vitro efficacy of SR-318, the LPS-stimulated TNF-a release in whole blood was determined. The results showed that this 5AP had an inhibition value of 97.7% at 10 mM (IC_50_ = 0.283 mM) in the assay, resulting in more effectiveness than the literature-known compounds [61].

Further studies focused on the exploration of the p38MAPK aC-out pocket showed that the hinge-binding motif of VPC00628 and SR-318 greatly enhanced the inhibitory activity compared with previously synthesised pyrazolyl-ureas. SARs extension of these reference derivatives led to the identification of Compound **18** (Figure 7) as a selective type-II inhibitor. Despite the moderate activity of the enzyme (IC_50_ = 14 nM), the crystallographic data provided valuable insights into the back-pocket interactions that were not observed in the SR-318/p38MPAK complex, thus indicating 5AP **18** as an alternative chemical tool with good cellular activity (PDB code: 6YK7; Figure 8). After demonstrating the excellent selectivity of **18**, human colon adenocarcinoma (HCT-15) cells were used to probe its efficiency on p38MPAKs. The Western blot analysis displayed the dose-dependent inhibition of p38 phosphorylation and increased phosphorylation of its downstream substrate HSP27. Furthermore, the new compound significantly inhibited TNF-a release with an IC_50_ value of 0.48 mM, a better result than the one obtained with the type-I inhibitor [62].

### 4.2. Anticancer/Antiproliferative 5APs

5APs show very interesting anticancer properties; for some compounds, the intracellular targets have been identified (e.g., Bruton kinase for the recently approved Pirtobrutinib) while for other derivatives, only the antiproliferative activity on different cancer cell lines or additional intracellular mechanism have been reported (see table in Section 6).

**Figure 9:**
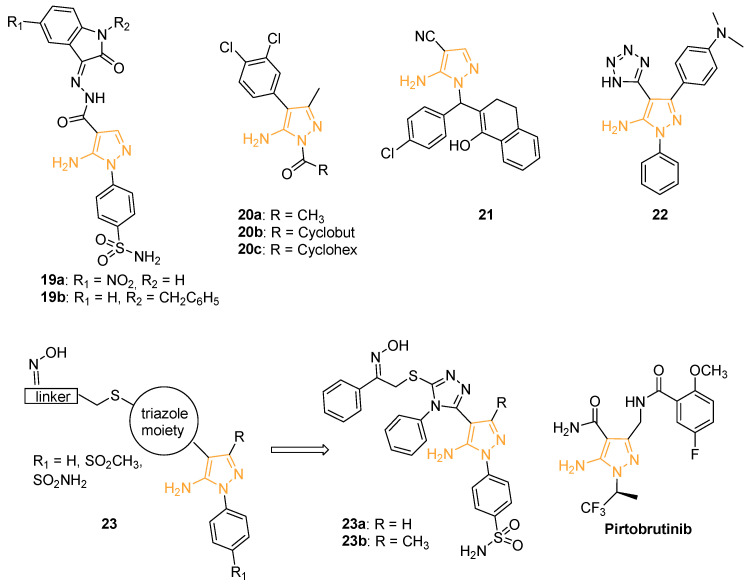
5APs as anticancer/antiproliferative compounds. The 5-aminopyrazole portion is highlighted in orange.

In 2015, Ibrahim and coworkers prepared a new series of benzenesulphonamide derivatives incorporating pyrazole and isatin moieties. A biological evaluation was carried out to assess the ability of the compounds to inhibit the metalloenzyme carbonic anhydrase (CA) and more precisely the human (h) isoforms hCA I, II (cytosolic), IX, and XII (transmembrane, tumour-associated enzymes). The 15 hCA isoforms are widely distributed within different tissues and are involved in many physiological and pathological conditions. In particular, the selective inhibition of hCA IX and XII produces significant antitumour and antimetastatic effects [63]. Derivatives **19a** and **19b** (Figure 9) inhibited hCA XII with a Ki of 5.4 nM and 7.2 nM, respectively. In particular, pyrazole **19a** with a 5-NO_2_ substitution on the isatin ring was found to be a selective inhibitor of hCA IX and hCA XII, being more active than the acetazolamide used as the reference drug. A docking simulation confirmed that the NO_2_ substituent present on **19a** participates in interactions with Asp132 within the hCA IX active site and with the Lys67 and Asp130 residues in hCA XII [64].

With the aim of identifying new chemical entities which can block the NF-kB cascade, Pippione, and coll. designed and synthesised a new series of 5APs that were biologically evaluated on four kinases involved in the NK-kB pathway (namely, IKKa, IKKb, IKKe, and NIK). In detail, 5APs **20a**–**c** (Figure 9) selectively inhibited NIK with an IC_50_ of 8.4, 2.9, and 3.3 nM, respectively. A gene-reported assay was used to measure NK-kB activation in human multiple myeloma EJM cells, constitutively characterised by high levels of nuclear NK-kB related to NIK activation, and in breast cancer cell lines (SKBr3 and MDA-MB-231) in which the constitutive activation of nuclear NK-kB is unrelated to NIK. Compounds **20a**–**c** seemed to inhibit the NK-kB activity in EJM cells (83.4–96.2% of inhibition at 25 mM), but not in SKBr3 and MDA-MB-231 cells, confirming their selective inhibitory activity for NIK [65].

In 2020, Hassan and his research group designed and synthesised a novel library of twenty amino-pyrazoles, pyrazolo-pyrimidines, and their fused analogues. The compounds were tested by the National Cancer Institute (NCI, Bethesda, Maryland, USA) at a fixed concentration of 10 mM on a panel of 60 different human cancer cell lines. 5-amino-1-((4-chlorophenyl)(1-hydroxy-3,4-dihydronaphthalen-2-yl)methyl)-1*H*-pyrazole-4-carbonitrile (compound **21**, Figure 9) showed the best cytotoxic activity with a cell proliferation inhibition higher than 90% on NCI-H23 (non-small cell lung cancer), HCT-15 (colon cancer), SF-295 (CNS cancer), NCI/ADR-RES (ovarian cancer), and DU-145 (prostate cancer) cells. As cyclooxygenase-2 (COX-2) inhibitors were largely reported to have antiproliferative activity against various cancer cells [66,67], docking simulations of **21** on COX-2 were carried out. Interestingly, the derivative displayed an affinity value of −7.86 kcal/mol and exhibited three hydrogen bonds with Lys137 and Gly45 side chains in the COX-2 active site [68].

Looking for new cytotoxic derivatives, Anwer and Sayed prepared a new series of heterocyclic compounds through microwave reactions. All the synthesised molecules were tested by an MTT assay at different concentrations (1–100 mg/mL) on breast cancer (MCF-7) and colorectal carcinoma (HCT-116) cells. Among the compounds of the library, 5AP **22** (3-(4-(dimethylamino)phenyl)-1-phenyl-4-(1*H*-tetrazol-5-yl)-1*H*-pyrazol-5-amine, Figure 9), bearing an additional tetrazole substituent on C4, displayed the best cytotoxic activity with an IC_50_ of 3.18 mM on HCT-116 and 4.63 mM on MCF-7 [69].

In the same year and for the same purpose, Fadaly and coworkers isolated a novel series of triazole/pyrazole hybrids analogues (compounds **23**, Figure 9) of COX-2 inhibitor Celecoxib endowed with a large spectrum of activity. The reported compounds were also tested by MTT assay for their antiproliferative activity on MCF-7, HCT-116, A549 (human lung cancer cell line), PC-3 (human prostate cancer cells), and F180 normal fibroblasts. The sulphamoyl derivatives **23a** and **23b** (Figure 9) were identified as the most active of the series with IC_50_ of 4.22 and 6.38 μM on A549, 5.33 and 3.67 μM on MCF-7, 3.46 and 2.28 μM on HCT-116, and 1.48 and 0.33 μM on PC-3, respectively. An investigation of the mechanism of action of these molecules revealed that they were able to block the cell cycle at the G2/M phase, with a downregulation of Bcl-2 gene expression and an up-regulation of Bax expression, evidencing a pro-apoptotic mechanism. Docking simulation studies indicated the Epidermal Growth Factor Receptor (EGFR) as a potential target of **23a** and **23b** whose oxime functionality would form two hydrogen bonds with Thr830A and Asp831A. Enzymatic and ELISA assays confirmed the ability of the compounds to interfere with p38MAPK and VEGFR-2 signalling pathways [70].

Very recently, Eli Lilly launched the new 5AP Pirtobrutinib (Jaypirca™, Figure 9), approved to treat mantle cell lymphoma (MCL), on the market [71,72,73,74,75,76]. In detail, this (S)-5-amino-3-(4-((5-fluoro-2-methoxybenzamido)methyl)phenyl)-1-(1,1,1-trifluoropropane-2-yl)-1*H*-pyrazole-4 carboxamide is a reversible inhibitor of Bruton Kinase (BTK), a nonreceptor tyrosine kinase, that represents a major therapeutic target for B-cell-driven malignancies. Different from previously approved covalent BTK inhibitors, innovative reversible BTK inhibitors, such as Pirtobrutinib, are characterised by limited off-target side effects and avoid the development of resistance mutations. These types of reversible BTK inhibitors have aroused great interest not only for the treatment of B cell malignancies but also for counteracting many autoimmune diseases [77]. Pirtobrutinib is currently being studied in 16 clinical trials (1 completed and 5 in phase III) for chronic lymphocytic leukaemia (CLL), small lymphocytic lymphoma (SLL), and mantle cell lymphoma (MCL) [78].

### 4.3. Antibacterial 5APs

In the last twenty years, a rise in the level of antimicrobial resistance among pathogens occurred due to the emergence of resistant strains of pathogenic bacteria and the increase in the number of antibiotics administered. Therefore, the development of new antibacterial compounds has become an urgent and mandatory worldwide need. For this purpose, 5APs were studied and showed more interesting results than their 3APs isomers (as compounds **2**, **3**). As reported below, in some cases, different 5APs revealed dual activity (anticancer and antibacterial action).

**Figure 10:**
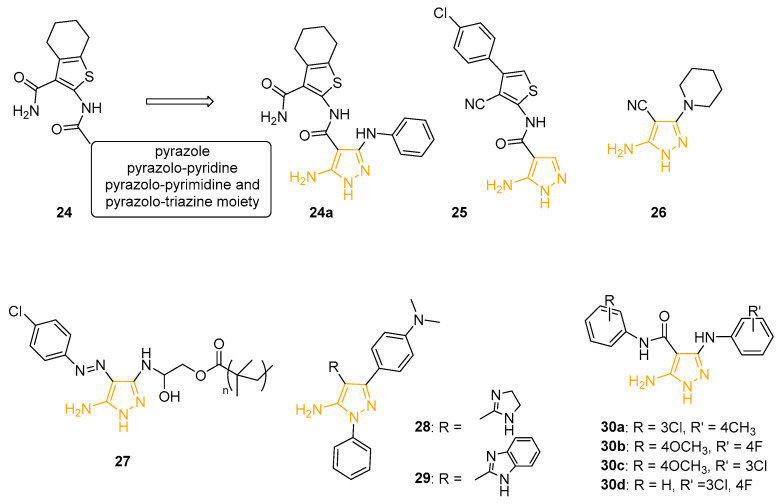
5APs with antibacterial properties. The 5-aminopyrazole portion is highlighted in orange.

In 2010, Gouda and coworkers prepared a novel series of heterocyclic derivatives in which 4,5,6,7-tetrahydro-benzo[*b*]thiophene-3-carboxamide scaffold was linked to pyrazole, pyrazolo-pyridine, pyrazolo-pyrimidine, or pyrazolo-triazine core (Compound **24** (Figure 10)) and used the agar diffusion technique to evaluate the antibacterial activity of the compounds on two bacterial strains (*B. theringiensis* and *K. pneumoniae*) and two fungi (*B. fabe* and *F. oxysporum*). 5AP **24a** (Figure 10) showed a larger inhibition zone diameter on Gram-positive *B. theringiensis* (24 mm) and Gram-negative *K. pneumoniae* (22 mm) than the reference drug Ampicillin (17 mm and 20 mm, respectively) [79].

In 2012, Behbehani and coworkers utilised 2-aminothiophenes as building blocks for the synthesis of new pyrazole, pyrimidine, quinoline, and pyridine-2-one derivatives. The isolated compounds were tested and evaluated as antimicrobial agents on a large panel of Gram-positive and Gram-negative bacteria strains and two fungi by well diffusion assay. 5AP **25** exhibited good activity against Gram-positive *B. subtilis* with an inhibition zone diameter of 7.3 ± 1.1 mm (Penicillin = 4.6 ± 1.1 mm). The compound also displayed moderate activity against fungi, such as *C. albicans* (6.6 ± 1.1 mm) and *S. serevisiae* (4 mm) [80].

Another class of 5APs with antibacterial properties was synthesised by Al-Adiwish in 2013. The antibacterial activity of the newly isolated compounds was tested using the agar diffusion technique at a concentration of 1 mg/mL on Gram-positive (*Staphylococcus aureus*, *Bacillus subtilis*, *Methicillin-resistant S. aureus*, *Staphylococcus epidermidis*, and *Enterococcus faecalis*) and Gram-negative (*Escherichia coli*, *Pseudomonas aeruginosa*, *Serratia marcescens*, and *Salmonella typhimurium*) bacteria. **26** (Figure 10) exhibited the best activity against *S. aureus* and *E. coli* (15 ± 0.58 mm and 15 ± 0.56 mm, respectively). The minimum inhibitory concentration (MIC) and minimum bactericidal concentration (MBC) were also determined: **26** showed a MIC of 16 mg/mL on *S. aureus* and 4 mg/mL on *E. coli*, whereas the MBC value results were 16 mg/mL on *S. aureus* and 8 mg/mL on *E. coli*. The derivative was also tested by an MTT assay on Vero cells to evaluate its in vitro cytotoxicity. Unfortunately, despite the safety of the compound (CC_50_ > 20 mg/mL), the selective index was too low to assess the selectivity of the molecule on the bacteria strains [81].

In 2018, Eldin reported the preparation, characterisation, and antimicrobial activity of new amphiphilic pyrazole-g-polyglycidyl methacrylate-based polymers. The goal of this study was to synthesise a new class of antimicrobial copolymers containing pendant amino groups in combination with hydrophobic residues to obtain new antimicrobial agents against a variety of clinically significant pathogens. In detail, 5AP **27** (Figure 10), characterised by an azo-substituent on the C4 position and devoid of antibacterial activity, was grafted to the epoxy rings of polyglycidyl methacrylate and poly (glycidyl methacrylate-co-methyl methacrylate) copolymers. The structure verification of the new polymers was performed using FT-IR and TGA analyses and new derivatives were tested against different Gram-positive and Gram-negative bacteria strains, showing various activity, particularly against Gram-negative pathogens. Pyrazole-g-polyglycidyl methacrylate showed the most potential antibacterial activities, followed by pyrazole-g-poly (glycidyl methacrylates-co-methyl methacrylate) copolymers. All these considerations open a new area for developing novel pyrazole-based antimicrobial agents [82].

Selected compounds from a heterocycle library synthesised by Anwer, including previously mentioned cytotoxic derivatives **22** (Figure 9) and 5APs **28** and **29** (Figure 10) displayed good antibacterial properties against both Gram-negative (*E. coli* and *P. aeruginosa*) and Gram-positive (*B. subtilis* and *S. aureus*) bacteria strains. Taking Ampicillin as a standard reference antibiotic, the activity index for each species was calculated. Pyrazole **22** exhibited the best results with activity index values ≥75% in all the analysed bacteria [69].

Recently, using a sequential ligand-based pharmacophore-based virtual screening followed by structure-based molecular docking, Indian researchers identified some 5APs endowed with anti-tubercular potential. In detail, 5-amino-3-((substitutes phenylamino)-*N*-(4-substituted phenylamino)-1*H*-pyrazole-4-carboxamides **30a**–**d** (Figure 10) showed potent anti-tubercular activity (MIC between 2.23 and 4.61 mM) compared to reference standard drugs and a lower cytotoxicity when tested on Vero cells. The preliminary SAR considerations suggested that the presence of the electron-withdrawing substituents at both R and R′ positions is beneficial for antibacterial activity, while electron-donating groups caused a remarkable reduction in potency. In addition, molecular docking studies suggested InhA, an enzyme involved in fatty acid synthesis and target for the development of novel antitubercular agents, as a possible target for the most potent derivatives [83].

### 4.4. Antimalarial 5APs

**Figure 11:**
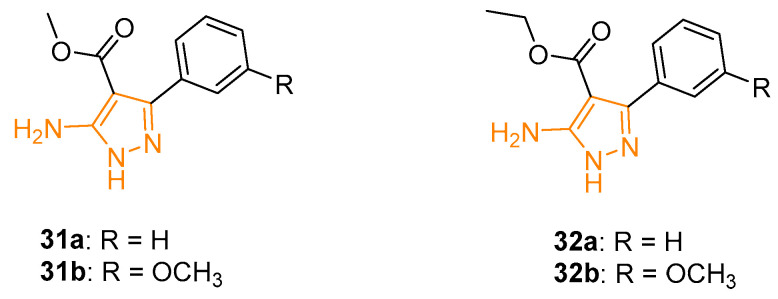
5APs reported as antimalarial agents. The 5-aminopyrazole portion is highlighted in orange.

The increasing prevalence of drug-resistant *Plasmodium falciparum* strains led to an urgent priority in the development of new antimalarial drugs. In this context, Dominguenz and coworkers, utilising a previously reported synthetic methodology, prepared a new series of APs with potential antimalarial activity. The activity of the novel derivatives was tested in vitro against *Plasmodium falciparum.* 5APs **31a** and **31b** (Figure 11) were the most active of the series with an IC_50_ of 0.149 mM and 0.150 mM, respectively. From a chemical point of view, the presence of the ester functionality in position 4 of the pyrazole ring seemed to be essential for the activity; indeed, its substitution with a nitrile group led to a loss of inhibition. Interestingly, the electronic effect of the substitution in the phenyl ring seemed to be important; in fact, the substitution with the methoxy group in the meta position increased the activity of the compounds, while the same group in position ortho or para resulted in a progressive loss of potency against the plasmodium [84].

A few years later, Verma and coworkers synthesised a series of pyrazole derivatives closely related to **31**, with an ethyl ester instead of a methyl one. All reported 5APs were tested in vitro against a chloroquine-resistant strain (FCBI) of *Plasmodium falciparum*. Not surprisingly, derivatives **32a** and **32b** (Figure 11) displayed the best IC_50_ with values of 0.149 mM and 0.150 mM, respectively, confirming the pivotal role of ester functionality and the meta substitution on the phenyl ring on C3 of the pyrazole ring. These two 5APs proved to be more effective than Pentamidine against *Leishmania donovani*, showing IC_50_ values of 0.132 mM and 0.168 mM, respectively. Interestingly, regarding pharmacological activity, the ortho and para substitution led to a loss of activity as previously evidenced for anti-Plasmodium action [85].

### 4.5. Anti-Inflammatory 5APs

**Figure 12:**
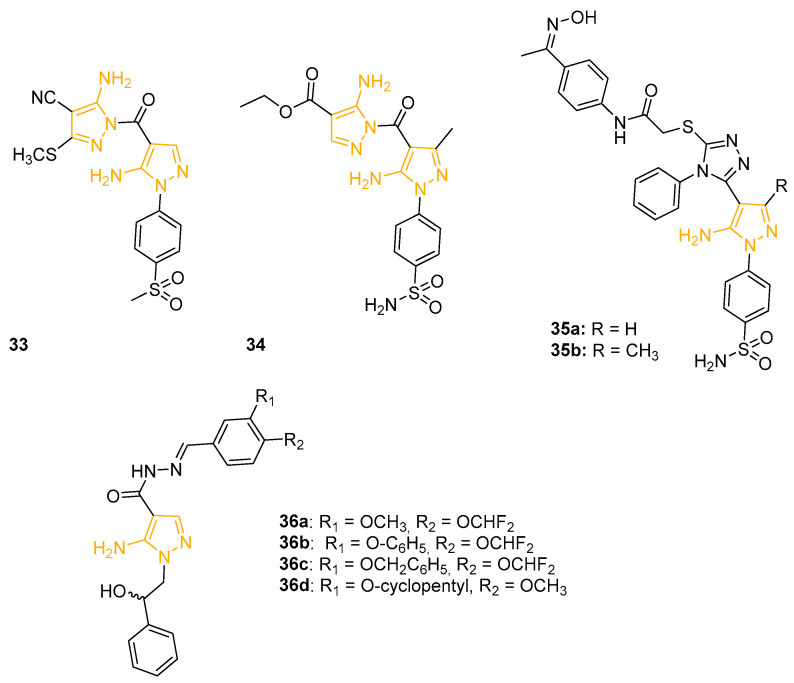
Structures of 5APs with anti-inflammatory activity. The 5-aminopyrazole portion is highlighted in orange.

In an effort to discover new selective COX-2 inhibitors without the ulcerogenic side effects of non-steroidal anti-inflammatory drugs (NSAIDs), Abdellatif and coworkers designed and prepared a new class of pyrazole derivatives possessing an amino or mehansulphonyl pharmacophore. All the compounds were preliminary tested against COX-1 and COX-2 by an in vitro colorimetric enzyme immunoassay (EIA). Furthermore, the COX-2 selectivity index (SI) was calculated [IC_50_ (COX-1)/IC_50_ (COX-2)] and compared to the reference drugs Indomethacin (non-selective COX inhibitor) and Celecoxib (selective COX-2 inhibitor). 5APs **33** and **34** (Figure 12), characterised by two linked 5AP portions, exhibited the best results with an IC_50_ on COX-2 of 39 nM and 34 nM, comparable to Celecoxib (IC_50_ = 45 nM), and a SI of 353.8 and 417.6, respectively. The in vivo carrageenan-induced rat paw oedema assay confirmed the in vitro data for **33** and **34**, orally administrated at a concentration of 50 mg/Kg. In addition, **33** and **34** displayed an oedema inhibition percentage of 96% and 87% after 5h, resulting in more effectiveness than the reference drug Celecoxib. In in vivo tests, the new derivatives caused a reduced number of ulcers when compared to Indomethacin and Celecoxib, with an ulcer index (UI) between 0.7 and 2 (UI Celecoxib = 2.7 and UI Indomethacin = 21.3), confirming their pharmaceutical attractiveness. A molecular docking study showed H-bond interactions between the SO_2_ groups of **33** and **34** and Phe504, Arg499, Tyr341, Ser516, and Arg106 amino acids, confirming the importance of the SO_2_ substituent on the 5AP scaffold to obtain COX-2 inhibition [86].

The following year, the same research group isolated a group of Celecoxib analogues linked to oxime moiety as nitric oxide donors, previously mentioned as anticancer agents [70]. The aim of the project was to synthesise novel anti-inflammatory NO-NSAID hybrids with selectivity for COX-2 and with a NO-donor moiety. 5APs **35a** and **35b** (Figure 12) showed higher activity in an in vitro COX-2 colorimetric assay (IC_50_ = 0.55 mM and 0.61 mM, respectively) compared to Celecoxib (IC_50_ = 0.83 mM). These two 5APs also displayed a good selectivity index (IC_50_ COX-1/IC_50_ COX-2) of 9.78 and 8.57, respectively, compared to 8.68 scored by the reference Celecoxib. To evaluate their in vivo anti-inflammatory activity, the derivatives were tested at a 50 mg/kg dose with the carrageenan-induced rat paw oedema assay. Pyrazole **35a** exhibited the best oedema inhibition percentage (91.11%), overcoming again the values of Celecoxib (86.66%). The ulcerogenic assay highlighted the reduced ulcerogenic effects of the newly synthesised molecules (ulcer indexes UI = 2.79–3.95) in comparison with Ibuprofen (UI = 20.25) and Celecoxib (UI = 2.93). The docking simulation of **35a** in the active site of COX-2 displayed that the compound would not form any H-bonds inside the COX-2 site. Furthermore, the percent of NO released from the derivatives was determined upon incubation in phosphate-buffered-saline. In detail, **35a** and **35b** exhibited a NO released percentage of 3.06% and 2.15%, respectively, which indicated a slow NO release.

More recently, to obtain new hybrid compounds potentially able to act on different targets involved in inflammation onset, Brullo and coworkers designed and synthesised a series of pyrazole and imidazo-pyrazole derivatives with differently decorated catechol moieties linked through an acylhydrazone chain. The inhibitory effect of the novel isolated molecules on reactive oxygen species (ROS) production on platelets and neutrophils was evaluated. 5APs **36a**, **36b**, and **36c** (Figure 12) displayed the most promising results, with IC_50_ values on ROS production inhibition in the low micromolar range with platelets, whereas derivative **36d** (Figure 12) showed a ROS production inhibition percentage of 68% on fMLP activated-neutrophils in flow cytometric analysis. The compound was also tested by enzymatic assay on phosphodiesterase enzymes PDE4B and PDE4D, intracellular enzymes mostly involved in neuroinflammation. The assay on **36d** resulted in an IC_50_ of 1.05 mM on PDE4D3 and an IC_50_ of 0.55 mM on PDE4B2, confirming the potential antioxidant and anti-inflammatory activity of this new chemotype [87]. Derivatives **36a**, **36b**, and **36c** were further investigated to evaluate their effect on several parameters indicative of oxidative status and their efficiency on aerobic metabolism. All three molecules seemed to strongly inhibit superoxide anion production, lipid peroxidation, and NADPH oxidase activity and almost restored the oxidative phosphorylation efficiency in platelets stimulated with thrombin, highlighting their potential protective effect against oxidative stress. These results were confirmed in endothelial cells in which the selected compounds showed a promising inhibition activity on H_2_O_2_-stimulated EA.hy926 cells [88].

### 4.6. 5APs with Other Pharmacological Activities

**Figure 13:**
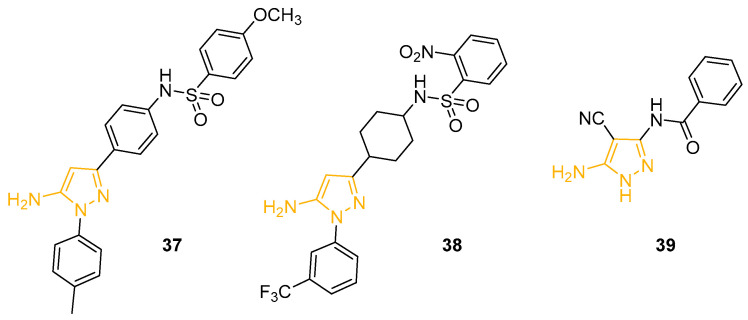
5APs reported as NPY Y5 inhibitors, insecticides, and anti-influenza agents. The 5-aminopyrazole portion is highlighted in orange.

In 2001, Kordik and collaborators prepared a novel series of 1,3-disubstituted-5APs with an affinity for the human neuropeptide Y (NPY) receptor Y5. NPY represents a powerful stimulant of food intake, implicated in obesity and eating; consequently, the antagonist of the NPY receptor Y5 could potentially provide new treatments for eating disorders. The synthesised 5APs, bearing a sulphonamide moiety, were tested for their binding affinity to NYP receptor Y5, using a transfected HEK293 cell line and measuring the competitive inhibition binding of ^125^I-PYY. Derivative **37** (Figure 13) displayed the best affinity in the first screening with an IC_50_ value of 15 nM [89].

Variation of the sulphonamide group led to inactive compounds, while the modification of the phenyl linker with a cyclohexyl group yielded pyrazole **38** (Figure 13), which showed a higher affinity for NPY Y5 than Fipronil (Figure 1) [90].

In 2020, Hebishy and collaborators described a new synthetic strategy to obtain novel benzamide-based 5APs as precursors of pyrazolo[1,5-*a*]pyrimidine and pyrazolo-[5,1-*c*][1,2,4]triazine derivatives; the derivatives were then tested as anti-influenza A agents active on subtype H5N1. The plaque reduction assay and the MTT cytotoxicity assay revealed 5AP **39** (Figure 13) as the most promising compound, with an inhibition percentage value of 66.67% at 0.125 mmol/mL concentration and an LD_50_ of 30 mmol/mL. Even though **39** resulted in less activity than the reference drug Zanamivir, it showed better potency than other reported bicycle analogues [91].

## 5. 3,5-Diaminopyrazoles (3,5-DAPs)

3,5-Diaminopyrazoles (3,5-DAPs) represent the most studied diaminopyrazole class and showed different biological properties including antiproliferative, antiviral, and antibacterial activities. Interestingly, as reported below, the majority of 3,5-DAPs with pharmacological activity are unsubstituted at the N1 position.

**Figure 14:**
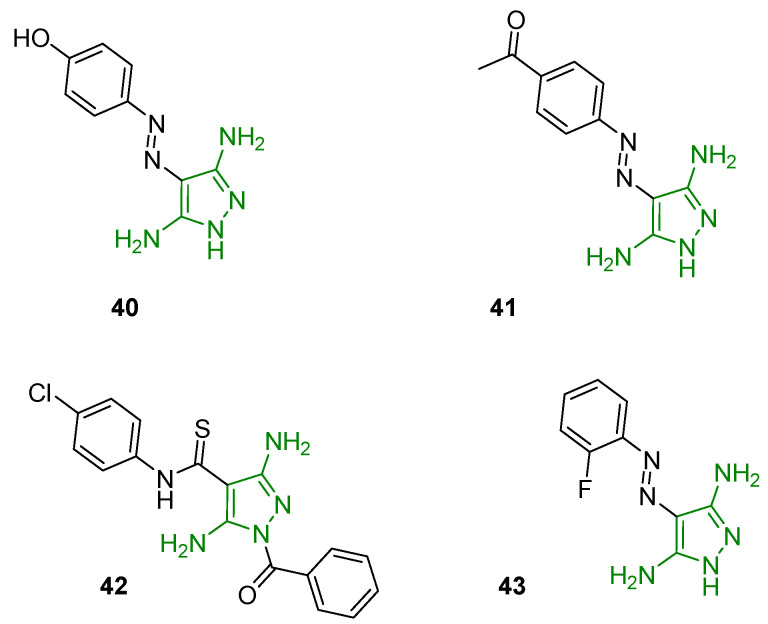
3,5DAPs reported as anticancer, antiviral, and antibacterial agents. The 3,5-diaminopyrazole portion is highlighted in green.

In 2006, Krystof and coworkers identified 4-arylazo-3,5-diamino-1*H*-pyrazoles active as ATP antagonists with potential selectivity for CDKs. Functional kinase assays confirmed the affinity toward CDKs, with a higher selectivity for the CDK9 isoform. The most promising derivative (compound **40**, Figure 14) displayed an IC_50_ of 3.5 mM on CDK2-cyclin E and additional investigations demonstrated that **40** acts as a competitive ATP inhibitor with a Ki value of 13.3 mM. Crystallographic analyses allowed the definition of the structural basis for the interaction of 3,5DAP and the CDK9 ATP binding pocket. Functional assays reported the ability of **40** to affect CDK9-related pathways including decreased phosphorylation of the retinoblastoma protein (pRb), inhibition of mRNA synthesis, and induction of the tumour suppressor p53protein. Finally, the evaluation of this new 3,5-DAP in an antiproliferation assay showed a reduced frequency of the S-phase of the cancer cell line HT-29 [92].

Recently, Ismail and coworkers designed and synthesised a new series of azo substituent 3,5-DAPs endowed with antiproliferative activity. The cytotoxic properties were evaluated in vitro against human breast cancer cells (MCF-7) and compound **41** (Figure 14) was seen to be the most active (IC_50_ of 26.86 mM). In vitro, a kinase assay individuated CDK2/cyclin E as a potential target of the newly synthesised derivatives [93].

Furthermore, a library of 3,5-diamino-*N*-aryl-1*H*-pyrazole-4-carbothioamides was identified as a potential class of HIV-1 inhibitors endowed with innovative mechanisms of action. The aim of the project was to synthesise new chemical entities able to inhibit different viral functions to provide a significant advantage against drug-resistant variants. All the novel DAPs were tested against RNase H activity, and derivative **42** (Figure 14) displayed good inhibition of viral replication and promising activity against both RNase H and RNA-dependent DNA polymerase (IC_50_ on RNase H of 7 mM). A docking simulation highlighted the binding of **42** to two RT (reverse transcriptase) pockets, one close to the polymerase catalytic site (RT-pocket 1) and one close to the RNase H catalytic site (RT-pocket 2), reinforcing the hypothesis of a dual-site inhibition. Moreover, these DAPs retained good inhibition potency against viral variants resistant to three non-nucleoside RT inhibitors (NNRTI) [94].

Finally, some 3,5-DAPs were recently reported as inhibitors of *P. Aeruginosa* biofilms. This type of infection was observed in several bacterial diseases such as cystic fibrosis-associated lung infections, chronic wound infections, catheter-associated urinary tract infections, and ventilator-associated pneumonia. Indeed, Jansens and coworkers synthesised sixty new derivatives and evaluated their cyclic di-GMP (c-di-GMP) reducing potency in the biofilms using the c-di-GMP monitor strain MTR235. Compound **43** (Figure 14), similar to **40** and **41** but bearing a fluorine group in the ortho position of the phenyl ring, was identified as the most active of the series, with a reduction in the c-di GMP level of 83%. These results suggested a stimulation of the bacteria phosphodiesterases, leading to a reduction of the cyclic nucleotide and the consequent degradation of the biofilm. The ortho-substitution of the phenyl ring as well as the primary amino groups on the pyrazole ring emerged to be essential for activity. The good activity and interesting pharmacokinetic profile of compound **43** laid a good foundation for the development of new antibiotics for the treatment of bacteria biofilms [95].

## 6. Conclusions

Due to its interesting pharmacological properties, the pyrazole nucleus has been extensively studied as a pharmacophore [96,97]; in particular, this heterocycle has been used to develop herbicides, agrochemicals, anticancer, anti-inflammatory, analgesic, antioxidant, anticonvulsant, antimicrobial, antimycobacterial, antiamoebic, antidepressant, hypotensive, and ACE inhibitors [98].

**Figure 15:**
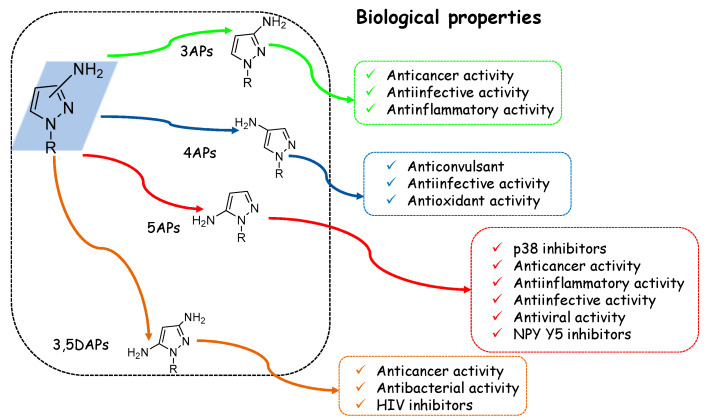
Schematic representation of the biological activity of 3APs, 4APs, 5APs, and 3,5DAPs.

As reported in Figure 15, the AP scaffold has also been deeply investigated from a medicinal chemistry point of view, particularly for its anti-inflammatory and anticancer activity [99,100].

Therefore, APs have been seen to be advantageous frameworks able to provide useful ligands for receptors or enzymes such as p38MAPK, different kinases involved in cancer progression, COX, and other targets important for bacterial and virus infections [101]. In particular, the most relevant results have been obtained for anticancer/anti-inflammatory compounds (Table 1), i.e., the recent approval of 5AP Pirtobrutinib, a reversible BTK inhibitor for the treatment of MCL, demonstrates the value of this scaffold for the development of new therapeutic agents.

In addition, the anti-infective (i.e., antimicrobial, antimycobacterial, antimalarial, and antiviral) properties of this chemotype are also worthy of note (Table 2), being able to overcome (myco)bacteria resistance to commonly used antibiotics.

Regarding SAR considerations, with the exception of the 4APs (not well investigated), it can be highlighted that:

(1) 3,5DAPs are biologically active when unsubstituted at the N1 position but embedded in the C-4 position;
(2) 5APs endowed with anticancer/anti-inflammatory properties are generally characterised at the N1 position by phenyl or phenethyl groups (compounds **19**–**23**, **33**–**36**);
(3) (3)5APs reported as p38 inhibitors (**RO3201195**, **VPC00628**, and **SR-318**) are characterised by a phenyl group at N1 and carbonil function of C4;
(4) 5APs characterised by antimalarial activity are unsubstituted at N1;
(5) 3APs and 5APS with anti-infective/antiviral activity could be substituted or unsubstituted at the N1 position.

In recent years, a large number of papers or reviews highlighting the design, synthesis, and biological evaluation of different classes of pyrazoles and many pyrazole-containing compounds have been reported in the literature, but an overview of APs (bearing a free amino group at the 3, 4, or 5 position) and their biological properties is still missing.

With the aim to fill this gap, the present review article focuses on aminopyrazole-based compounds in different therapeutic fields, with particular attention to the design and structure-activity relationship (SAR) aspects of each class of compounds, to provide better correlation among different currently ongoing research.

## Figures and Tables

**Table 1:** Summary of the anticancer/anti-inflammatory activity of APs.

AP Class	Cpd	Biological Activity	Target(s)	Ref
3APs	5	Anticancer	Tubulin	[33]
	6	Anticancer and antioxidant	/	[35]
	7	Antiproliferative and anti-mitotic	/	[38]
	8	Antiproliferative	CG interrupting site	[39]
	9	Anti-inflammatory	MK2	[40]
	10	Anti-inflammatory	GSK-3β	[41]
	11	Antiproliferative	/	[42]
4APs	15	Anticancer	/	[48]
	16	Antioxidant	/	[49]
5APs	RO3201195	Anti-inflammatory	p38α	[56]
	17	Anti-inflammatory	p38α	[59]
	VPC00628	Anti-inflammatory	p38α	[60]
	SR-318	Anti-inflammatory	p38α	[61]
	18	Anti-inflammatory	p38α	[62]
	19	Anticancer	hCA XII	[64]
	20	Anticancer	NF-κB	[65]
	21	Antiproliferative	COX-2	[68]
	22	Antiproliferative	/	[69]
	23	Antiproliferative	EGFR	[70]
	33	Anti-inflammatory	COX-2	[86]
	34	Anti-inflammatory	COX-2	[86]
	35	Anti-inflammatory	COX-2	[70]
	36	Antioxidant	PDE4	[87]
3,5DAPs	40	Anticancer	CDK9	[92]
	41	Antiproliferative	CDK2/cyclin	[93]

**Table 2:** Summary of the anti-infective activity of APs.

AP Class	Cpd	Biological Activity	Target(s)	Ref
3APs	1	Antibacterial	*S. aureus* (G+)	[29]
	2	Antibacterial	*S. aureus* (G+) and *E. coli* (G−)	[30]
	3	Antibacterial and antifungal	*B. subtilis* (G+), *S. pneumoniae* (G+), *E. coli* (G−), *A. flavus*, *S. racemosum*, and *G. candidum*	[31]
	4	Antiviral	HIV-1 reverse transcriptase	[32]
5APs	22	Antibacterial	*B. subtilis* (G+), *S. aureus* (G+), *E. coli* (G−), and *P. aereuginosa* (G−)	[69]
	24	Antibacterial and antifungal	*B. theringiensis* (G+), *K. pneumoniae* (G−), *B. fabe*, and *F. oxysporum*	[79]
	25	Antibacterial and antifungal	*B. subtilis* (G+), *C. albicans*, and *S. cerevisiae*	[80]
	26	Antibacterial	*S. aureus* (G+) and *E. coli* (G−)	[81]
	27	Antibacterial	Gram-negative strains	[82]
	28	Antibacterial	*B. subtilis* (G+), *S. aureus* (G+), *E. coli* (G−), and *P. aereuginosa* (G−)	[69]
	29	Antibacterial	*B. subtilis* (G+), *S. aureus* (G+), *E. coli* (G−), and *P. aereuginosa* (G−)	[69]
	30	Anti-tubercular	InhA	[83]
	31	Antimalarial	*P. falciparum*	[84]
	32	Antimalarial and antiprotozoal	*P. falciparum* and *L. donovani*	[85]
	39	Antiviral	Influenza A (H5N1)	[91]
3,5DAPs	42	Antiviral	HIV-1 RNase H	[94]
	43	Antibacterial	*P. aereuginosa* (G−)	[95]

## Data Availability

Not applicable.

## Abbreviations

ACE	Angiotensin-converting enzyme
AD	Alzheimer’s Disease
ADME	Absorption, distribution, metabolism, and excretion
Aps	Aminopyrazoles
ATP	Adenosine triphosphate
BTK	Bruton kinase
c-di-GMP	Cyclic dimeric guanosine monophosphate
CDKs	Cyclin-dependent kinases
CLL	Chronic Lymphocytic Leukemia
COX	Cyclo-Oxygenase
DNA	Deoxyribonucleic acid
EGFR	Epidermal growth factor receptor
ELISA	Enzyme-linked immunosorbent assay
ERK	Extracellular signal-regulated kinase
fMLP N-	N-Formylmethionyl-leucyl-phenylalanine
GABA	Gamma-aminobutyric acid
GSK	Glycogen synthase kinase
hCA	Human carbonic anhydrase
HDF	Human dermal fibroblasts
HIV	Human immunodeficiency virus
HWB	Human whole blood
IKK	Ikappa Kinase
IL	Interleukin
JNK	Jun N-terminal kinase
LPS	Lipopolysaccharide
MAPKs	Mitogen-activated protein kinases
MBC	Minimum bactericidal concentration
MCL	Mantle cell lymphoma
MIC	Minimum inhibitory concentration
MK2	Mitogen-activated protein kinase-2
MRSA	Methicillin-resistant Staphylococcus aureus
MSSA	Methicillin-sensitive Staphylococcus aureus
MTT	[3-(4,5-Dimethylthiazol-2-yl)-2,5-Diphenyltetrazolium Bromide]
NADPH	Nicotinamide adenine dinucleotide phosphate
NCI	National cancer institute
NF-κB	Nuclear factor kappa B
NIK	NF-κB inducing kinase
NNRTI	Non-nucleoside RT inhibitor
NO	Nitric oxide
NPY	Neuropeptide Y
NSAIDs	Non-steroidal anti-inflammatory drugs
PDE	Phosphodiesterase
PTZ	Pentylene-tetrazole-induced seizure
ROS	Reactive oxygen species
RT	Reverse transcriptase
SARs	Structure-activity relationships
SLL	Small lymphocytic lymphoma
TFOs	Triplex forming oligonucleotide
TNFα	Tumour necrosis factor-alpha
VEGFR	Vascular endothelial growth factor
WS	Werner syndrome
